# Cytotoxic Compounds from the Saudi Red Sea Sponge *Xestospongia testudinaria*

**DOI:** 10.3390/md14050082

**Published:** 2016-04-26

**Authors:** Ali A. El-Gamal, Shaza M. Al-Massarani, Lamiaa A. Shaala, Abdulrahman M. Alahdald, Mansour S. Al-Said, Abdelkader E. Ashour, Ashok Kumar, Maged S. Abdel-Kader, Wael M. Abdel-Mageed, Diaa T. A. Youssef

**Affiliations:** 1 Department of Pharmacognosy, College of Pharmacy, King Saud University, P.O. Box 2457, Riyadh 11451, Saudi Arabia; aelgamal00@yahoo.com or aelgamel@ksu.edu.sa (A.A.E.-G.); salmassarani@ksu.edu.sa (S.M.A.-M.); msalsaid@ksu.edu.sa (M.S.A.-S.); waelcognosy@yahoo.com (W.M.A.-M.); 2 Department of Pharmacognosy, Faculty of Pharmacy, Mansoura University, El-Mansoura 35516, Egypt; 3 Natural Products Unit, King Fahd Medical Research Center, King Abdulaziz University, Jeddah 21589, Saudi Arabia; lshalla@kau.edu.sa or lamiaelnady@yahoo.com; 4 Suez Canal University Hospital, Suez Canal University, Ismailia 41522, Egypt; 5 Department of Clinical Pharmacy, Faculty of Pharmacy, King Abdulaziz University, Jeddah 21589, Saudi Arabia; amalahdal@kau.edu.sa; 6 Department of Pharmacology and Toxicology, College of Pharmacy, King Saud University, P.O. Box 2457, Riyadh 11451, Saudi Arabia; aeashour@yahoo.com; 7 Vitiligo Research Chair, College of Medicine, King Saud University, Riyadh 11451, Saudi Arabia; aknirankari@gmail.com; 8 Department of Pharmacognosy, College of Pharmacy, Sattam Bin Abdulaziz University, Al-kharj 11942, Saudi Arabia; mpharm101@hotmail.com; 9 Department of Pharmacognosy, Faculty of Pharmacy, Assiut University, Assiut 71526, Egypt; 10 Department of Natural Products, Faculty of Pharmacy, King Abdulaziz University, Jeddah 21589, Saudi Arabia

**Keywords:** Red Sea sponge, *Xestospongia testudinaria*, xestosterol fatty acids esters, brominated acetylenic fatty acids, cancer cell lines, cytotoxic activity

## Abstract

Bioassay-guided fractionation of the organic extract of the Red Sea sponge *Xestospongia testudinaria* led to the isolation of 13 compounds including two new sterol esters, xestosterol palmitate (**2**) and xestosterol ester of l6′-bromo-(7′*E*,11′*E*,l5′*E*)-hexadeca-7′,11′,l5′-triene-5′,13′-diynoic acid (**4**), together with eleven known compounds: xestosterol (**1**), xestosterol ester of 18′-bromooctadeca-7′*E*,9′*E*-diene-7′,15′-diynoic acid (**3**), and the brominated acetylenic fatty acid derivatives, (5*E*,11*E*,15*E*,19*E*)-20-bromoeicosa-5,11,15,19-tetraene-9,17-diynoic acid (**5**), 18,18-dibromo-(9*E*)-octadeca-9,17-diene-5,7-diynoic acid (**6**), 18-bromooctadeca-(9*E*,17*E*)-diene-7,15-diynoic acid (**7**), 18-bromooctadeca-(9*E*,13*E*,17*E*)-triene-7,15-diynoic acid (**8**), l6-bromo (7*E*,11*E*,l5*E*)hexadeca-7,11,l5-triene-5,13-diynoic acid (**9**), 2-methylmaleimide-5-oxime (**10**), maleimide-5-oxime (**11**), tetillapyrone (**12**), and nortetillapyrone (**13**). The chemical structures of the isolated compounds were accomplished using one- and two-dimensional NMR, infrared and high-resolution electron impact mass spectroscopy (1D, 2D NMR, IR and HREIMS), and by comparison with the data of the known compounds. The total alcoholic and *n*-hexane extracts showed remarkable cytotoxic activity against human cervical cancer (HeLa), human hepatocellular carcinoma (HepG-2), and human medulloblastoma (Daoy) cancer cell lines. Interestingly, the dibrominated C_18_-acetylenic fatty acid (**6**) exhibited the most potent growth inhibitory activity against these cancer cell lines followed by Compounds **7** and **9**. Apparently, the dibromination of the terminal olefinic moiety has an enhanced effect on the cytotoxic activity.

## 1. Introduction

Marine sponges (phylum Porifera) are among the oldest and simplest animals, which grow in every ocean and have a great capacity to withstand extreme temperatures and pressures [1]. They are filter feeders, and their bodies are full of pores and channels that allows water to circulate through them [2,3]. Moreover, they are well-known for their production of secondary metabolites that constitute an effective defense mechanism against foreign predators [2,3]. So far, about 8000 species of Porifera, inhabiting different marine and freshwater ecosystems, have been described. Since the beginning of the exploration of marine natural products in the 1970s, the investigation of the secondary metabolites of *Xestospongia* sponges (family Petrosiidae), commonly known as barrel sponges, have been carried out successively in several regions around the world [4]. They have been recognized as rich sources of different chemical classes, such as isoquinoline, macrocyclic quinolizidines, pyridoacridine alkaloids, quinones, sterols, brominated polyacetylenic acids, and esters [4]. Previous investigations on the chemistry and pharmacology of genus *Xestospongia* have shown that its crude extracts and isolated compounds displayed remarkable bioactivities, such as anti-inflammatory, antioxidant, immunomodulatory, cytotoxicity, antimicrobial, insecticidal, HIV protease inhibition, cardiotonic, vasodilatation, and antiplasmodial activities [5,6,7,8,9]. The currently investigated species, Xestospongia testudinaria, has been the source of indole alkaloids, sterols, sterol esters, and brominated polyunsaturated fatty acids (BPUFAs) [4,10,11].

As part of our continued interest in identifying potential marine-derived bioactive compounds for treatment of contemporary diseases such as cancer and infectious diseases [12,13,14,15], we thoroughly investigated the chemical constituents and cytotoxic activity of the Red Sea sponge *Xestospongia testudinaria*. In this paper, we report about the purification and identification of thirteen compounds including two new xestersterol esters (**2** and **4**) with eleven known compounds. In addition, the cytotoxic activities of the compounds against three cancer cell lines will be evaluated.

## 2. Results and Discussion

**Figure 1:**
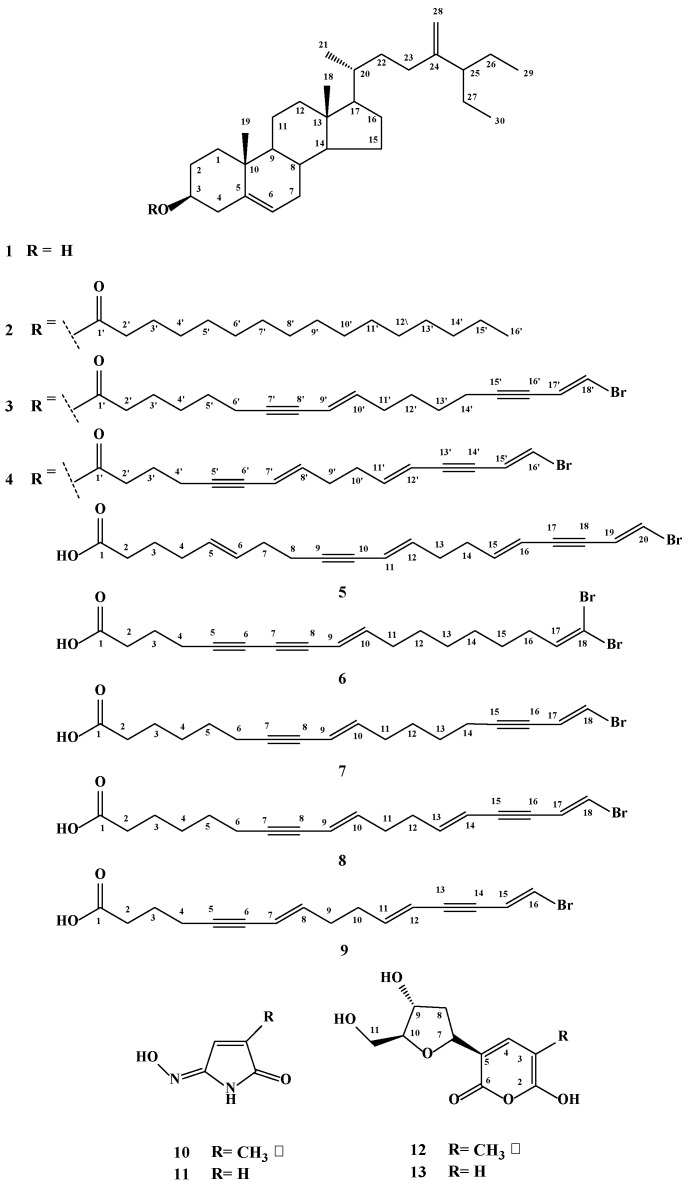
Structures of Compounds **1**–**13**.

Compound **2** (Figure 1) was isolated as a white powder. Its HREIMS displayed [M]^+^ at 664.6158 consistent with the molecular formula C_46_H_80_O_2_ and seven degrees of unsaturation. The intense IR absorption band at 1735 cm^−1^ indicated the presence of a carbonyl ester, which was also evident from a quaternary carbon signal, in ^13^C NMR, at 172.1 ppm (Table 1 and Table 2). The ^13^C NMR data of **2** (Table 1) possesses the signals for a basic steroid nucleus with 30 carton atoms (xestosterol type) as established by the similarity of its ^1^H and ^13^CNMR data with those of xestosterol (**1**) [16]. The significant differences between Compounds **1** and **2** lie in the downfield shift of H-3/C-3 from δ_H/C_ 3.53/71.8 in **1** to δ_H/C_ 4.55/73.6 in **2**, and an upfield shift of C-4 from δ_C_ 42.2 ppm in **1** to δ_C_ 38.2 ppm in **2**. These differences, together with the presence of a carbonyl ester signal at δ_C_ 172.1 ppm in **2**, suggested the esterification of **2** at C-3. Based on evidence obtained from HR-EI-MS, ^1^H, and ^13^C NMR (Table 1 and Table 2), the ester moiety at C-3 was determined to be a long chain fatty acid (palmitic acid) [17]. The ^13^C NMR and DEPT experiments revealed the presence of a total of 46 carbons ascribed as 30 carbons for the xestosterol skeleton, while the remaining 16 carbons were assigned to the long chain fatty acid moiety. The ^1^HNMR spectrum (Table 2) showed a signal at δ_H_ 0.79, assigned to the terminal methyl of the long chain fatty acid, beside a long chain methylene at δ_H_ 1.29 integrated for 22 protons. The terminal methyl carbon appeared, in ^13^CNMR, at δ_C_ 14.1 ppm, while the methylene carbons of the long chain moiety appeared at δ_C_ 34.4, 25.1, 31.9, and 22.7, assigned to C-2’, C-3’, C-14’, and C-15’, respectively, and from 29.1 to 29.8 (11 CH_2_) for C-4’–C-13’. The complete assignment for all protons and carbons for both the steroid nucleus and the long chain fatty acid moiety was achieved by the aid of ^1^H–^1^H COSY and HSQC experiments. A careful interpretation of the HMBC spectra allowed the final structural elucidation of **2**, whereas two and three bond correlations were observed from H-4 to C-1’, C-2, C-4, C-6, and C-10; from H-2 to C-1 and C-3; from H-13’ to C-14’ and C-15’; and from H-2‘ to C-1’, C-3’, and C-4’. Thus, Compound **2** was deduced as xestosterol-3 palmitate and is reported here as a new natural product.

Compound **4** (Figure 1) was isolated as a yellowish brown powder. HR-FAB-MS showed [M]^+^ at 728.4168, consistent with the molecular formula C_46_H_65_BrO_2_ with 14 degrees of unsaturation. IR showed bands at 2240, 1731, and 1469 cm^−1^, assigned to an acetylenic group, an ester group, and a terminal methylene group, respectively. The ^13^C NMR (Table 1) and DEPT experiments showed a total of 46 carbon atoms, 30 of which were assigned to a xestosterol nucleus similar to Compounds **1**–**3**. Xestosterol has previously been isolated and partially chemically synthesized from the same sponge [16]. On the other hand, careful analysis of the remaining 16 carbons revealed a close similarity between signals of the brominated polyacetylenic acid **9**, except for the free carboxylic acid carbon (C-1, δ_C_ 179.8), which was shifted to 173.1 ppm in **4**, confirming the presence of an ester moiety. The *E* configurations at C7’/C8’, C11’/C12’, and C15’/C16’ were secured from the large coupling constants b (15.8–16.2 Hz). Like **2**, the downfield shift of H-3, compared to **1**, and a ^3^*J* cross peak correlation, in the HMBC experiment, from H-3 (δ_H_ 4.57) to C-1’ (ester carbonyl at δ_C_ 173.1) justified the esterification’s being at position three. Additional cross peak correlations were observed in the HMBC experiment from H-1 to C-3, C-5, and C-19. The above data proved that Compound **4** is a new ester of xestosterol-3-(l6′-bromo-7′*E*,11′*E*,l5′*E*-hexadeca-7′,11′,l5′-triene-5′,13′-diynoic acid) isolated here for the first time from a natural source. It is worth mentioning that xestosterol esters with brominated acetylenic fatty acid moieties have rare occurrences in marine sponges with few published reports [10,11,18].

Using a combination of 1D, 2D NMR spectra (Table 1 and Table 2) and HREIMS determinations and by comparing their spectral data with those in the literature, the known compounds were identified as xestosterol (24-methylene,26,27-dimethylcholest-5-en-3β-ol) (**1**) [16], xestosterol ester of 18′-bromo-9′*E*,17′*E*-octadecadiene-7′,15′-diynoic acid (**3**) [10], (5*E*,11*E*,15*E*,19*E*)-20-bromoeicosa-5,11,15,19-tetraene-9,17-diynoic acid (**5**) [19], 18,18-dibromo-9*E*-octadeca-9,17-diene-5,7-diynoic acid (**6**) [20], 18-bromooctadeca-9*E*,17*E*-diene-7,15-diynoic acid (**7**) [21], 18-bromooctadeca-9*E*,13*E*,17*E*-triene-7,15-diynoic acid (**8**) [11], l6-bromo-7*E*,11*E*,l5*E*-hexadeca-7,11,l5-triene-5,13-diynoic acid (**9**) [20], 2-methyl maleimide-5-oxime (**10**) [22], maleimide-5-oxime (**11**) [23], tetillapyrone (**12**) [24], and nortetillapyrone (**13**) (Figure 1) [24].

The isolated compounds were evaluated for their antitumor activity against three cancer cell lines—human hepatocellular carcinoma (HepG-2), human medulloblastoma (Daoy), and human cervical cancer (HeLa) cells—using an MTT assay, as described previously [25], using dasatinib as a positive reference drug. Five concentrations (0–50 µg/mL) of each compound were prepared and incubated with each cell line, and the survival fraction curves were obtained to calculate the concentration that produced 50% cell growth inhibition (IC_50_).

As shown in Table 3, the total ethanolic extract of *X. testudinaria*, *n*-hexane fraction, and Compound **6** exhibited broad spectrum inhibition of all tested cancer cell lines. In this context, the potency can be arranged in the following descending order: Compound **6** > *n*-hexane fraction > the total ethanolic extract on human cervical cancer (HeLa) and Daoy cells, while on HepG-2 cells, **6** > the total ethanolic extract > *n*-hexane fraction. Compounds **7** and **9** showed moderate selectivity against HeLa and Daoy cells. Compound **7** was more potent than **9**, since the IC_50_ of **7** was 30.38 and 23.1 µg/mL on HeLa and Daoy cells, respectively, compared to **9**, which exhibited IC_50_ values of 44.41 and 24.57 µg/mL on HeLa and Daoy cells, respectively. With regard to the sensitivity of cancer cells to the compounds, the Daoy cell line appeared to be the most sensitive towards the tested compounds, followed by HepG-2 and HeLa cell lines. Structure-activity relationships are proposed by comparing the IC_50_ values of the isolated brominated polyacetylenic fatty acids. The results suggest that the cytotoxic activity was dramatically enhanced by the presence of an extra bromine atom as in Compound **6**.

With regard to the brominated compounds (**5**, **6**, **7** and **9**), the dibrominated compound (**6**) showed stronger cytotoxic activity than the monobrominated ones. On the other hand, the presence of a conjugated triple bond in Compound **6** may have contributed to the improved activity.

## 3. Materials and Methods

### 3.1. General Experimental Procedure

The IR spectra were recorded on JASCO 320-A spectrometers, while the ^1^H and ^13^C NMR spectra were recorded at the NMR Unit at the College of Pharmacy, Prince Sattam Bin Abdulaziz University, on an Ultra Shield Plus 500 MHz (Bruker) spectrometer operating at 500 MHz for proton and 125 MHz for carbon, respectively. The chemical shift values are reported in δ (ppm) relative to the TMS as an internal standard. 2D-NMR experiments (COSY, HSQC, HMBC, and NOESY) were obtained using a standard Bruker program. A HR-EI-MS, JEOL JMS-700, was used for accurate mass determination. Electron impact mode of ionization was used, keeping ionization energy at 70 ev. Resolution was set up to 10 k. A direct probe was used with a temperature ramp setting—an initial temperature of 50 °C, rising at a rate of 32 °C per minute, and a final temperature set up to 350 °C. Pre-coated silica gel TLC plates were used. The absorbance was read on a microplate reader (ELX 800, Bio-Tek Instruments, Winooski, VT, USA) at 549 nm. MTT (3-(4,5-dimethylthiazol-2-yl)-2,5-diphenyltetrazolium bromide) was acquired from Sigma Aldrich (St Louis, MO, USA). DMEM/high glucose FBS and penicillin/streptomycin were purchased from Thermo Fisher Scientific. (Waltham, MA, USA).

### 3.2. Animal Material

The sponge was collected by hands using SCUBA diving from Ghurab Reef in the Red Sea at Jazan, Saudi Arabia between 15 and 30 meters in May 2013. The sponge was frozen immediately after collection and then freeze-dried to provide the dry material.

The sponge was identified as *Xestospongia testudinaria* by Prof. Rob van Soest at the Naturalis Biodiversity Center at Leiden in The Netherlands. A sample of the sponge is reserved in the collections of the Naturalis Biodiversity Center under the number RMNH Por. 9176. Another voucher specimen was placed in the Red Sea Marine Invertebrates Collection, Faculty of Pharmacy, King Abdulaziz University under the code No. DY-KSA-12. A complete description of the sponge has been previously published [5].

### 3.3. Extraction and Isolation

The freeze-dried sponge (750 g) was extracted with 96% ethanol (3 × 2 L) at room temperature. The combined alcohol extracts were filtered and evaporated under reduced pressure using a rotatory evaporator at 38 °C to produce 115 g of the alcohol extract. When methanol (500 mL) was added to the dried ethanolic extract, a precipitate was formed and collected (26 g). Part of this precipitate was washed repeatedly with different organic solvents to yield a pure compound (**1**). The other part was applied over a silica gel column with *n*-hexane/acetone in gradient elution mode to yield Compounds **2**–**4**. On the other hand, the methanol soluble part of the ethanolic extract (88 g) was suspended in 40% ethanol (1 L) and successively partitioned with 500-mL portions of *n*-hexane, dichloromethane, and *n-*butanol to afford the corresponding fractions.

The *n-*hexane fraction (19 g) was chromatographed on a silica gel column (35 mm i.d. × 350 mm) and eluted with a *n-*hexane/acetone gradient. TLC monitoring allowed the constitution of 3 main fractions (A–C). Fraction A, eluted by 10% acetone, was chromatographed on a Sephadex LH-20 column using 10% H_2_O/MeOH for elution to afford both **7** and **8**; Fraction B was subjected to a RP 18 column with gradient H_2_O/CH_3_CN elution to yield three subfractions (I–III). Direct crystallization of subfraction II afforded **9** (20% H_2_O/CH_3_CN). Subfraction I and III eluted by 30% H_2_O was further purified separately by chromatotron (Harrison Research, Palo Alto, California, CA, USA) (centrifugal TLC) (silica gel 60, 0.04–0.06 mm, 1 mm using MeOH/CHCl_3_ solvent system, to give **5** and **6**.

The CH_2_Cl_2_ (1.5 g) and *n-*BuOH (2.2 g) fractions were combined due to the similarity on TLC, part of which (0.9 g) was further purified by column chromatography using a MeOH/DCM gradient to give, after TLC examination, five fractions (1–5). Fractions 1, 2, and 3 eluted with 2% MeOH/CH_2_Cl_2_ yielded, after further purification, Compounds **10**–**12**, respectively. Fraction 4, also eluted with 2% MeOH/CH_2_Cl_2_, was separately purified by chromatotron using 12% MeOH/CH_2_Cl_2_ to give Compound **13**.

### 3.4. Spectral Data of the Compounds

*Xestosterol palmitate* (**2**). White powder; HR-EI-MS *m/z* 664.6158 [M]^+^ for C_46_H_80_O_2_; IR (KBr) *v*_max_ 2934, 2867, 2212, 2190, 1734, 1463, 956, 886 cm^−1^. ^1^H NMR (CDCl_3_, 500 MHz): δ*_H_* 1.05, 1.71 (1H each, m, H-1), 1.58, 1.76 (1H each, m, H-2), 4.45, (1H, m, H-3), 2.48 (2H, br t, *J* = 12Hz, H-4), 5.33 (1H, br s, H-6), 1.36, 1.92 (1H each, m, H-7), 1.46 (1H, m, H-8), 0.90 (1H, m, H-9), 1.45 (2H, m, H-11), 1.18, 2.07 (1H each, m, H-12), 0.96 (1H, m, H-14), 1.07, 1.56 (1H, m, H-15), 1.31, 1.89 (1H each, m, H-16), 1.15 (1H, m, H-17), 0.67 (3H, s, H-18), 1.01(3H, s, H-19), 1.47 (1H, m, H-20), 0.97, (3H, d, *J* = 7.5Hz, H-21), 1.37, 1.79 (2H, m, H-22), 1.98, 2.19 (2H, m, H-23), 1.89 (1H, m, H-25), 1.47 (2H, m, H-26), 1.46 (2H, m, H-27), 4.65, 4.73 (2H, br s, H-28), 0.82, (3H, t, *J* = 7.3Hz, H-29), 0.82 (3H, t, *J* = 7.3 Hz, H-30), ^1^HNMR of side chain fatty acid: see Table 2, ^13^CNMR: see Table 1.

*Xestosterol ester of l6-bromo-7E,11E,l5E-hexadeca-7,11,l5-triene-5,13-diynoic acid* (**4**). Yellowish brown powder; HR-FAB-MS *m/z* 728.4168 [M]^+^ for C_46_H_65_BrO_2_. IR (KBr) *v*_max_ 2240, 1731, 1469 cm^−1^. ^1^H NMR (CDCl_3_, 500 MHz): δ*_H_* 4.57 (1H, m, H-3), 4.57 (1H, d, *J* = 0.9Hz, H-6), 0.65 (3H, s, H-18), 0.99, (3H, s, H-19), 0.91, (3H, d, *J* = 6.5 Hz, H-21), 4.65, 4.72 (2H, br s, H-28), 0.76, (3H, t, *J* = 7.3 Hz, H-29), 0.76 (3H, t, *J* = 7.3Hz, H-30). ^1^HNMR of side chain fatty acid: see Table 2, ^13^CNMR: see Table 1.

*2-Methyl maleimide-5-oxime* (**10**). ^13^CNMR (DMSO*-d_6_*, 125 MHz): δ*_C_* 164.93 (CO-2), 151.47 (C-5), 137.70 (*C*H-4), 107.67 (C-3), 11.75 (CH_3_).

*Maleimide-5-oxime* (**11**). ^13^CNMR (DMSO*-d_6_*): δ*_C_* 164.93 (C-2), 151.4 (C-5), 137.7 (C-4), 107.6 (C-3).

*Tetillapyrone* (**12**). ^13^C NMR (MeOD): δ_C_ 152.4 (C-2), 111.6 (C-3), 138.2 (C-4), 111.6 (C-5), 166.5 (C-6), 86.3 (C-7), 41.2 (C-8), 72.2 (C-9), 88.8 (C-10), 62.7 (C-11), 12.5 (CH_3_).

*Nortetillapyrone* (**13**). ^13^C NMR (MeOD): δ_C_ 152.3 (C-2), 102.8 (C-3), 142.6 (C-4), 102.8 (C-5), 166.3 (C-6), 86.7 (C-7), 41.3 (C-8), 72.3 (C-9), 89.0 (C-10), 62.9 (C-11).

### 3.5. Evaluation of the Antiproliferative Activity of the Compounds Using MTT Assay

The three utilized tumor cell lines were human cervical cancer (HeLa), human hepatocellular carcinoma (HepG-2) and human medulloblastoma (Daoy) cells. HeLa and HepG-2 cells were cultured in DMEM/high glucose supplemented with 10% FBS, 2 mM of l-glutamine, and 1% penicillin/streptomycin. Daoy cells were cultured in DMEM/F12 supplemented with 10% FBS, 2 mM of l-glutamine, and 1% penicillin/streptomycin.

All isolated compounds were evaluated at the Cell Culture Laboratory, College of Pharmacy, King Saud University, in a three-cell line-one concentration (50 mg/mL) anticancer assay against the aforementioned cell lines, adapting the method described by Al-Salahi *et al.* 2014 [25].

The dose response curves of the compounds affecting ≥50% inhibition in one-dose prescreening for each cell line were established with concentrations of 50, 25, 12.5, 6.25, 3.125, and 1.56 µg/mL, and the concentrations causing 50% cell growth inhibition (IC_50_) were calculated. The cytotoxic activity of the anticancer drug dasatinib [26] against the tested cell lines was examined at the same concentrations of the tested compounds.

## 4. Conclusions

In conclusion, bioassay-directed fractionation of the active fractions of the organic extracts of the Red Sea sponge *Xestospongia testudinaria* afforded thirteen compounds including two new xestosterol esters (**2** and **4**) together with several known compounds (**1**, **2**, **5**–**13**). The identification of the compounds was achieved by analysis of the NMR and MS data of the compounds and by comparison with the literature. The compounds showed different cytotoxic activities towards the three tested cancer cell lines.

## Figures and Tables

**Table 1:** ^13^C NMR data of Compound **1** and steroid part of Compounds **2**–**4** (125 MHz, CDCl_3_).

Position	δ_C_			
	1	2	3	4
1	37.3	37.1	37.0	37.0
2	28.2	28.2	28.2	28.2
3	71.8	73.6	73.7	73.8
4	42.2	38.2	38.2	38.2
5	140.7	138.6	139.7	139.7
6	121.7	122.5	122.6	122.6
7	31.5	31.9	31.9	31.9
8	31.9	32.8	31.9	31.9
9	50.2	50.2	50.2	50.2
10	35.8	36.1	36.6	36.6
11	21.1	21.1	21.0	21.0
12	39.8	39.7	39.7	39.7
13	42.4	41.3	42.3	42.3
14	56.8	56.7	56.7	56.7
15	24.3	24.3	24.3	24.3
16	29.4	29.4	29.3	29.3
17	56.0	56.0	56.0	56.0
18	11.9	11.9	11.9	11.9
19	19.4	19.3	19.3	19.3
20	35.8	35.8	35.8	35.8
21	18.8	18.8	18.8	18.8
22	34.4	34.4	34.5	34.4
23	31.5	31.4	31.9	31.9
24	152.6	151.3	152.5	152.6
25	50.1	50.0	50.0	50.0
26	26.4	26.5	26.3	26.4
27	26.3	26.3	26.4	26.3
28	108.8	108.9	108.8	108.8
29	12.0	12.0	12.0	12.0
30	12.1	12.1	12.1	12.0

**Table 2:** Partial ^1^HNMR data of **2** and **4** and ^13^C NMR data of fatty acids moieties of **2**–**9**.

Position	δ_H_ (m, *J* in Hz)		δ_C_							
	2	4	2	3	4	5	6	7	8	9
1′			172.1	173.0	173.1	179.5	179.5	180.4	180.2	179.8
2′	2.20 (t, 7.5)	2.50 (t, 7.5)	34.4	31.9	31.9	38.7	32.7	34.0	34.0	32.8
3′	1.60 (m)	1.85 (m)	25.1	24.6	24.3	23.4	23.2	24.2	24.2	23.6
4′	1.29 (m)	2.39 (t, 6.0)	29.1	28.0	18.8	28.9	18.9	27.9	27.9	18.7
5′	1.29 (m)		29.3	27.8	89.1	132.7	81.9	27.7	27.7	87.6
6′	1.29 (m)		29.6	19.3	79.1	124.5	66.3	19.2	19.2	79.9
7′	1.29 (m)	5.42 (d, 15.8)	29.7	92.7	111.0	38.7	72.8	88.5	88.9	110.8
8′	1.29 (m)	5.95 (m)	29.8	79.2	141.2	18.6	74.4	79.3	79.1	141.8
9′	1.29 (m)	2.22 (m)	29.8	110.3	32.5	87.2	108.6	110.3	110.3	32.4
10′	1.29 (m)	2.22 (m)	29.8	142.5	31.9	79.7	148.3	142.6	142.6	31.9
11′	1.29 (m)	6.10 (m)	29.6	32.4	144.3	110.7	33.0	32.4	31.9	144.3
12′	1.29 (m)	5.55 (dd, 15.9, 1.5)	29.4	28.5	110.0	141.9	28.7	28.4	32.5	110.0
13′	1.29 (m)		29.3	28.3	90.4	31.9	28.7	28.3	144.3	90.4
14′	1.29 (m)		31.9	19.2	84.9	32.4	27.7	19.2	110.0	84.9
15′	1.40 (m)	6.25 (dd, 16.2, 2.2)	22.7	88.6	117.7	144.3	28.4	92.8	90.4	117.7
16′	0.80 (t, 6.9)	5.60 (d, 14.0)	14.1	77.3	117.8	110.0	33.2	77.5	84.9	117.8
17′				117.9		90.4	138.8	117.9	117.7	
18′				117.1		84.9	88.6	117.1	117.5	
19′						117.7				
20′						117.9				

**Table 3:** Cytotoxic activity of *X. testudinaria* extracts and isolated compounds ^1^.

Test Sample	HeLa Cell		HepG-2		Daoy	
	% Inhibition ^2^	IC_50_^3^	% Inhibition ^2^	IC_50_^3^	% Inhibition ^2^	IC_50_^3^
EtOH extract	83.35	35.07	91.24	23.45	91.6	23.31
DCM + *n*-BuOH	36.45	NT ^4^	8.37	NT ^4^	36.45	NT ^4^
*n*-Hexane	91.28	33.7	89.31	30.2	90.5	20.74
**1**	35.78	NT ^4^	46.25	NT ^4^	34.07	NT ^4^
**2**	7.98	NT ^4^	14.72	NT ^4^	0	NT ^4^
**5**	4.17	NT ^4^	2.09	NT ^4^	0	NT ^4^
**6**	87.98	23.85	89.33	17.72	87.02	15.72
**7**	67	30.38	18.4	NT ^4^	77.56	23.1
**9**	58.61	44.41	45.23	NT ^4^	71.58	24.57
Dasatinib	56.4	16.22	89.67	6.91	90.36	9.2

^1^ Compounds **10**–**13** were evaluated before and were weakly cytotoxic, while **3**, **4**, and **8** were not tested due to lack of materials. ^2^ The percent of cell survival inhibition at 50 µg/mL, compared to control. ^3^ IC_50s_ are expressed in (µg/mL). ^4^ NT: Not tested. HeLa: human cervical cancer; HepG-2: human hepatocellular carcinoma; Daoy: human medulloblastoma.
